# *N*′-[3-Cyano-4-(4-fluoro­phen­yl)-6-meth­oxy-4*H*-benzo[*h*]chromen-2-yl]-*N*,*N*-dimethyl­methanimidamide

**DOI:** 10.1107/S1600536813005503

**Published:** 2013-03-02

**Authors:** Al-anood M. Al-dies, Mohamed A. Al-Omar, Abd El-Galil E. Amr, Ahmed M. El-Agrody, Seik Weng Ng, Edward R. T. Tiekink

**Affiliations:** a Chemistry Department, Faculty of Science, King Khalid University, Abha 61413, PO Box 9004, Saudi Arabia; b Pharmaceutical Chemistry Department, College of Pharmacy, King Saud University, Riyadh 11451, Saudi Arabia; c Drug Exploration & Development Chair (DEDC), College of Pharmacy, King Saud University, Riyadh 11451, Saudi Arabia; d Applied Organic Chemistry Department, National Research Center, Dokki 12622, Cairo, Egypt; e Department of Chemistry, University of Malaya, 50603 Kuala Lumpur, Malaysia; f Chemistry Department, Faculty of Science, King Abdulaziz University, PO Box 80203 Jeddah, Saudi Arabia

## Abstract

In the title compound, C_24_H_20_FN_3_O_2_, despite the 4*H*-pyran ring having a flattened half-chair conformation [the methine C atom lies 0.257 (3) Å above the plane of the remaining atoms with an r.m.s. deviation of 0.0295 Å], the 14 non-H atoms of the 4*H*-benzo[*h*]chromene residue are approximately coplanar (r.m.s. deviation = 0.081 Å). The benzene ring is nearly perpendicular to this plane [dihedral angle = 76.18 (10)°], but the planar (r.m.s. deviation = 0.033 Å) dimethyl­methanimidamide substituent is coplanar [dihedral angle = 1.96 (12)°]. In the crystal, centrosymmetric dimeric aggregates arise from C—H⋯N inter­actions, and these are connected into supra­molecular layers in the *ab* plane by C—H⋯π and π–π [inter­centroid (central C_6_ ring)⋯(outer C_6_ ring)^i^ distance = 3.8564 (14) Å] inter­actions.

## Related literature

For background to synthetic aspects of benzochromene derivatives, see: El-Agrody *et al.* (2011 ▶); Sabry *et al.* (2011 ▶). For biological inter­est in these derivatives, see: Kidwai *et al.* (2010 ▶); Singh *et al.* (2010 ▶); Vukovic *et al.* (2010 ▶); Abd-El-Aziz *et al.* (2007 ▶). For a closely related structure, see: Al-Dies *et al.* (2012 ▶).
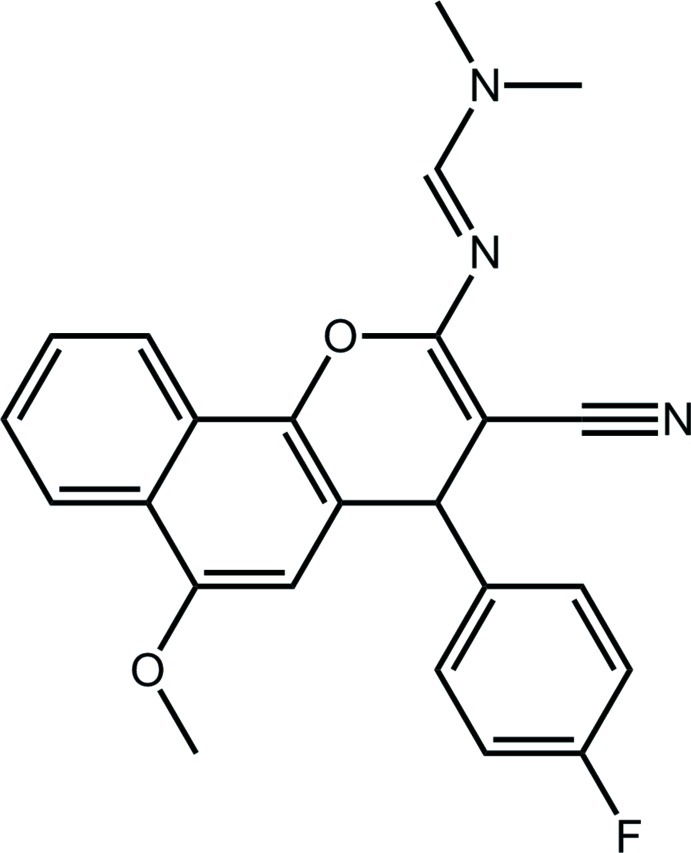


## Experimental

### 

#### Crystal data

C_24_H_20_FN_3_O_2_
*M* *_r_* = 401.43
Triclinic, 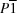

*a* = 8.8438 (8) Å
*b* = 11.0887 (12) Å
*c* = 11.8001 (13) Å
α = 66.054 (10)°
β = 83.684 (8)°
γ = 75.946 (9)°
*V* = 1025.85 (18) Å^3^
*Z* = 2
Mo *K*α radiation
μ = 0.09 mm^−1^
*T* = 295 K
0.30 × 0.20 × 0.10 mm

#### Data collection

Agilent SuperNova Dual diffractometer with an Atlas detector
Absorption correction: multi-scan (*CrysAlis PRO*; Agilent, 2011 ▶) *T*_min_ = 0.982, *T*_max_ = 1.000
9814 measured reflections
4745 independent reflections
2729 reflections with *I* > 2σ(*I*)
*R*_int_ = 0.034

#### Refinement

*R*[*F* ^2^ > 2σ(*F* ^2^)] = 0.058
*wR*(*F* ^2^) = 0.179
*S* = 1.04
4745 reflections
273 parameters
H-atom parameters constrained
Δρ_max_ = 0.20 e Å^−3^
Δρ_min_ = −0.24 e Å^−3^

### 

Data collection: *CrysAlis PRO* (Agilent, 2011 ▶); cell refinement: *CrysAlis PRO*; data reduction: *CrysAlis PRO*; program(s) used to solve structure: *SHELXS97* (Sheldrick, 2008 ▶); program(s) used to refine structure: *SHELXL97* (Sheldrick, 2008 ▶); molecular graphics: *ORTEP-3 for Windows* (Farrugia, 2012 ▶) and *DIAMOND* (Brandenburg, 2006 ▶); software used to prepare material for publication: *publCIF* (Westrip, 2010 ▶).

## Supplementary Material

Crystal structure: contains datablock(s) global, I. DOI: 10.1107/S1600536813005503/hg5296sup1.cif

Structure factors: contains datablock(s) I. DOI: 10.1107/S1600536813005503/hg5296Isup2.hkl

Supplementary material file. DOI: 10.1107/S1600536813005503/hg5296Isup3.cml

Additional supplementary materials:  crystallographic information; 3D view; checkCIF report

## Figures and Tables

**Table 1:** Hydrogen-bond geometry (Å, °) *Cg*1, *Cg*2 and *Cg*3 are the centroids of the C18–C23, C2–C7 and C1,C2,C7–C10 rings, respectively.

*D*—H⋯*A*	*D*—H	H⋯*A*	*D*⋯*A*	*D*—H⋯*A*
C23—H23⋯N3^i^	0.93	2.62	3.542 (3)	171
C5—H5⋯*Cg*1^ii^	0.93	2.79	3.670 (3)	159
C15—H15*B*⋯*Cg*2^iii^	0.96	2.93	3.732 (3)	142
C16—H16*C*⋯*Cg*3^iii^	0.96	2.91	3.589 (3)	129

Symmetry codes: (i) 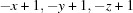; (ii) 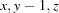; (iii) 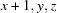.

**Fig. 1.**
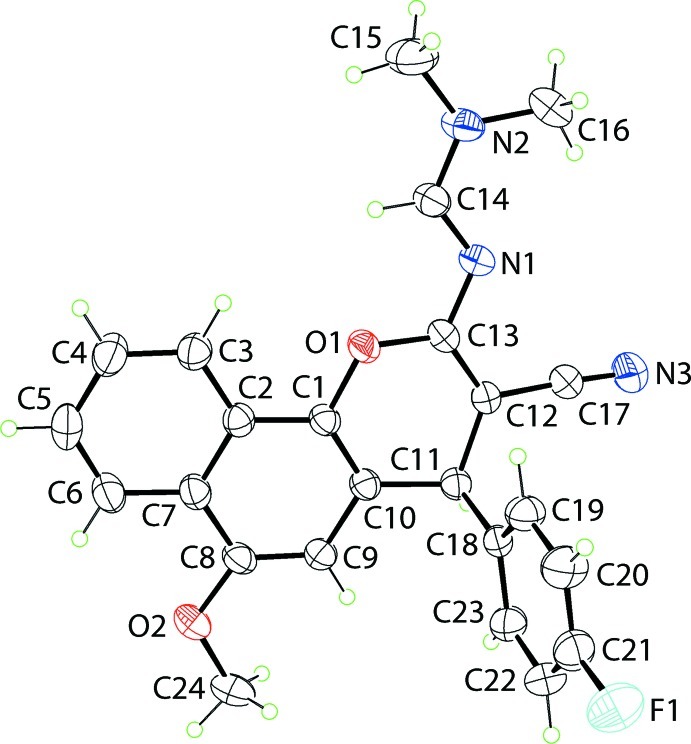
The molecular structure of (I) showing the atom-labelling scheme and displacement ellipsoids at the 35% probability level.

**Fig. 2.**
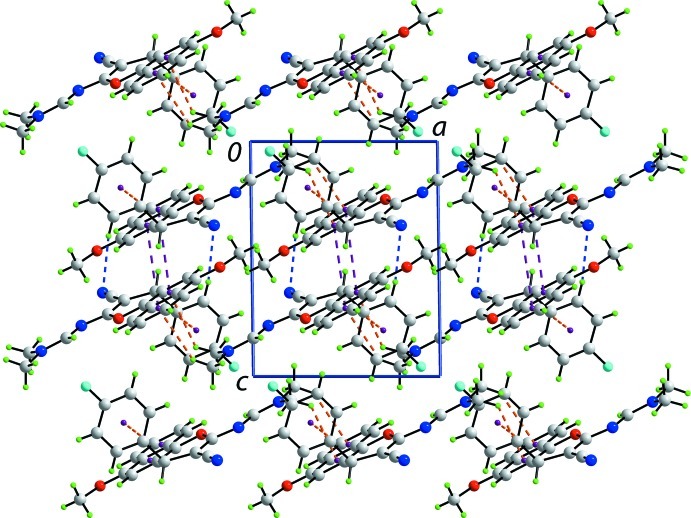
View in projection down the b axis of the crystal packing in (I). The C—H···N, C—H···π and π—π interactions are shown as blue, orange and purple dashed lines, respectively.

## supplementary crystallographic information

### Comment

Benzochromene derivatives have recently received intensified interest due to their synthetic (El-Agrody *et al.*, 2011; Sabry *et al.*, 2011) and pharmaceutical importance (Kidwai *et al.*, 2010; Singh *et al.*, 2010; Vukovic *et al.*, 2010). In continuation of our interest in the chemical and pharmacological properties of 4*H*-chromene and fused 4*H*-chromene derivatives (Abd-El-Aziz *et al.*, 2007), the X-ray crystal structure of the title compound, (I), was determined.

In (I), Fig. 1, the 4*H*-pyran ring approximates a half-chair conformation with the methine-C11 atom lying 0.257 (3) Å out of the least-squares plane defined by the remaining atoms (O1,C1,C10,C12 and C13) which have a r.m.s. deviation of 0.0295 Å. Despite this, the 14 non-hydrogen atoms comprising the 4*H*-benzo[*h*]chromene fused ring system approximate a plane with a r.m.s. deviation of 0.081 Å. The benzene ring is approximately perpendicular to this plane, forming a dihedral angle of 76.18 (10)°. The atoms (N1,N2,C14–C16) comprising the dimethylmethanimidamide residue are planar (r.m.s. deviation = 0.033 Å) and this is co-planar with the 4*H*-benzo[*h*]chromene residue; dihedral angle = 1.96 (12)°. Finally, the methoxy group is co-planar with the ring to which it is connected as manifested in the C24—O2—C8—C9 torsion angle of 3.7 (3)°. The overall structure resembles closely that reported recently for the parent amine (Al-Dies *et al.*, 2012).

In the crystal packing, C—H···N interactions, Table 1, lead to centrosymmetric dimeric aggregates. These are linked into layers in the *ab* plane by C—H···π, Table 1, and π—π [inter-centroid (C1,C2,C7–C10)···(C2—C7)^i^ distance = 3.8564 (14) Å, angle of inclination = 0.07 (11)° for *i*: 1 - *x*, *-y*, 1 - *z*] interactions, Fig. 2.

### Experimental

A mixture of 2-amino-4-(4-fluorophenyl)-6-methoxy-4*H*-benzo[*h*]chromene-3-carbonitrile (0.01 mol), dimethylformamide-dipentylacetal (DMF-DPA) (0.01 mol) and benzene (30 ml) was refluxed for 3 h. The solvent was removed under reduced pressure and the resulting solid was recrystallized from benzene to give the title compound; *M*.pt: 513–514 K.

### Refinement

The C-bound H atoms were geometrically placed (C—H = 0.93–0.98 Å) and refined as riding with *U_iso_*(H) = 1.2–1.5*U_eq_*(C).

### Crystal data

**Table:**

C_24_H_20_FN_3_O_2_	*Z* = 2
*M**_r_* = 401.43	*F*(000) = 420
Triclinic, *P*1	*D*_x_ = 1.300 Mg m^−^^3^
Hall symbol: -P 1	Mo *K*α radiation, λ = 0.71073 Å
*a* = 8.8438 (8) Å	Cell parameters from 2074 reflections
*b* = 11.0887 (12) Å	θ = 2.9–27.5°
*c* = 11.8001 (13) Å	µ = 0.09 mm^−^^1^
α = 66.054 (10)°	*T* = 295 K
β = 83.684 (8)°	Prism, light-brown
γ = 75.946 (9)°	0.30 × 0.20 × 0.10 mm
*V* = 1025.85 (18) Å^3^	

### Data collection

**Table:**

Agilent SuperNova Dual diffractometer with an Atlas detector	4745 independent reflections
Radiation source: SuperNova (Mo) X-ray Source	2729 reflections with *I* > 2σ(*I*)
Mirror monochromator	*R*_int_ = 0.034
Detector resolution: 10.4041 pixels mm^-1^	θ_max_ = 27.6°, θ_min_ = 2.9°
ω scan	*h* = −11→11
Absorption correction: multi-scan (*CrysAlis PRO*; Agilent, 2011)	*k* = −14→10
*T*_min_ = 0.982, *T*_max_ = 1.000	*l* = −15→15
9814 measured reflections	

### Refinement

**Table:**

Refinement on *F*^2^	Primary atom site location: structure-invariant direct methods
Least-squares matrix: full	Secondary atom site location: difference Fourier map
*R*[*F*^2^ > 2σ(*F*^2^)] = 0.058	Hydrogen site location: inferred from neighbouring sites
*wR*(*F*^2^) = 0.179	H-atom parameters constrained
*S* = 1.04	*w* = 1/[σ^2^(*F*_o_^2^) + (0.0687*P*)^2^ + 0.1794*P*] where *P* = (*F*_o_^2^ + 2*F*_c_^2^)/3
4745 reflections	(Δ/σ)_max_ < 0.001
273 parameters	Δρ_max_ = 0.20 e Å^−^^3^
0 restraints	Δρ_min_ = −0.24 e Å^−^^3^

### Special details

**Table:**

Geometry. All e.s.d.'s (except the e.s.d. in the dihedral angle between two l.s. planes) are estimated using the full covariance matrix. The cell e.s.d.'s are taken into account individually in the estimation of e.s.d.'s in distances, angles and torsion angles; correlations between e.s.d.'s in cell parameters are only used when they are defined by crystal symmetry. An approximate (isotropic) treatment of cell e.s.d.'s is used for estimating e.s.d.'s involving l.s. planes.
Refinement. Refinement of *F*^2^ against ALL reflections. The weighted *R*-factor *wR* and goodness of fit *S* are based on *F*^2^, conventional *R*-factors *R* are based on *F*, with *F* set to zero for negative *F*^2^. The threshold expression of *F*^2^ > σ(*F*^2^) is used only for calculating *R*-factors(gt) *etc*. and is not relevant to the choice of reflections for refinement. *R*-factors based on *F*^2^ are statistically about twice as large as those based on *F*, and *R*- factors based on ALL data will be even larger.

### Fractional atomic coordinates and isotropic or equivalent isotropic displacement parameters (Å^2^)

**Table:**

	*x*	*y*	*z*	*U*_iso_*/*U*_eq_	
F1	0.10342 (19)	0.85738 (18)	0.04207 (18)	0.1067 (7)	
O1	0.71783 (15)	0.23009 (15)	0.24647 (13)	0.0480 (4)	
O2	0.17217 (17)	0.07626 (18)	0.46549 (17)	0.0682 (5)	
N1	0.91690 (18)	0.34311 (18)	0.21651 (16)	0.0462 (4)	
N2	1.1331 (2)	0.2837 (2)	0.10802 (18)	0.0584 (5)	
N3	0.8000 (2)	0.5769 (2)	0.3565 (2)	0.0720 (6)	
C1	0.5773 (2)	0.1989 (2)	0.30364 (19)	0.0406 (5)	
C2	0.5430 (2)	0.0847 (2)	0.29533 (19)	0.0429 (5)	
C3	0.6411 (3)	0.0088 (2)	0.2340 (2)	0.0520 (6)	
H3	0.7331	0.0337	0.1965	0.062*	
C4	0.6024 (3)	−0.1006 (3)	0.2292 (2)	0.0659 (7)	
H4	0.6684	−0.1499	0.1887	0.079*	
C5	0.4646 (3)	−0.1394 (3)	0.2847 (3)	0.0698 (7)	
H5	0.4393	−0.2142	0.2806	0.084*	
C6	0.3676 (3)	−0.0688 (3)	0.3445 (2)	0.0617 (7)	
H6	0.2766	−0.0962	0.3815	0.074*	
C7	0.4025 (2)	0.0459 (2)	0.3515 (2)	0.0476 (5)	
C8	0.3038 (2)	0.1228 (2)	0.4135 (2)	0.0484 (5)	
C9	0.3424 (2)	0.2318 (2)	0.4185 (2)	0.0463 (5)	
H9	0.2769	0.2803	0.4599	0.056*	
C10	0.4819 (2)	0.2725 (2)	0.36114 (18)	0.0407 (5)	
C11	0.5173 (2)	0.3992 (2)	0.36151 (18)	0.0408 (5)	
H11	0.5020	0.3962	0.4461	0.049*	
C12	0.6864 (2)	0.4009 (2)	0.32420 (19)	0.0418 (5)	
C13	0.7733 (2)	0.3255 (2)	0.26505 (19)	0.0421 (5)	
C14	0.9972 (2)	0.2654 (2)	0.1639 (2)	0.0515 (6)	
H14	0.9582	0.1941	0.1654	0.062*	
C15	1.2168 (3)	0.2017 (4)	0.0420 (3)	0.0856 (9)	
H15A	1.1658	0.1296	0.0559	0.128*	
H15B	1.3218	0.1644	0.0714	0.128*	
H15C	1.2183	0.2568	−0.0452	0.128*	
C16	1.1981 (3)	0.3944 (3)	0.1012 (3)	0.0690 (7)	
H16A	1.1416	0.4334	0.1569	0.103*	
H16B	1.1899	0.4618	0.0181	0.103*	
H16C	1.3057	0.3612	0.1243	0.103*	
C17	0.7526 (2)	0.4977 (2)	0.3405 (2)	0.0510 (6)	
C18	0.4058 (2)	0.5249 (2)	0.27590 (19)	0.0415 (5)	
C19	0.4420 (3)	0.5885 (2)	0.1521 (2)	0.0554 (6)	
H19	0.5369	0.5554	0.1211	0.066*	
C20	0.3412 (3)	0.6998 (3)	0.0731 (3)	0.0704 (7)	
H20	0.3675	0.7421	−0.0100	0.084*	
C21	0.2032 (3)	0.7458 (3)	0.1193 (3)	0.0668 (7)	
C22	0.1591 (3)	0.6859 (3)	0.2398 (3)	0.0699 (8)	
H22	0.0621	0.7186	0.2684	0.084*	
C23	0.2623 (2)	0.5750 (2)	0.3191 (2)	0.0582 (6)	
H23	0.2349	0.5338	0.4021	0.070*	
C24	0.0645 (3)	0.1533 (3)	0.5229 (3)	0.0758 (8)	
H24A	−0.0230	0.1117	0.5552	0.114*	
H24B	0.0291	0.2434	0.4628	0.114*	
H24C	0.1147	0.1566	0.5893	0.114*	

### Atomic displacement parameters (Å^2^)

**Table:**

	*U*^11^	*U*^22^	*U*^33^	*U*^12^	*U*^13^	*U*^23^
F1	0.0857 (11)	0.0803 (13)	0.1207 (16)	0.0159 (9)	−0.0371 (10)	−0.0162 (11)
O1	0.0466 (8)	0.0539 (10)	0.0540 (9)	−0.0222 (7)	0.0144 (6)	−0.0293 (8)
O2	0.0510 (9)	0.0675 (12)	0.0898 (13)	−0.0299 (8)	0.0205 (8)	−0.0305 (10)
N1	0.0380 (9)	0.0529 (11)	0.0474 (11)	−0.0126 (8)	0.0044 (7)	−0.0191 (9)
N2	0.0426 (10)	0.0811 (15)	0.0584 (13)	−0.0176 (10)	0.0095 (8)	−0.0347 (11)
N3	0.0654 (13)	0.0720 (16)	0.0951 (18)	−0.0265 (12)	0.0034 (11)	−0.0440 (14)
C1	0.0399 (10)	0.0404 (12)	0.0400 (11)	−0.0130 (9)	0.0035 (8)	−0.0129 (9)
C2	0.0453 (11)	0.0383 (12)	0.0425 (12)	−0.0096 (9)	−0.0024 (9)	−0.0127 (9)
C3	0.0568 (13)	0.0457 (13)	0.0532 (14)	−0.0128 (10)	0.0043 (10)	−0.0195 (11)
C4	0.0809 (17)	0.0533 (16)	0.0714 (18)	−0.0162 (13)	0.0074 (13)	−0.0337 (14)
C5	0.0837 (18)	0.0531 (16)	0.088 (2)	−0.0263 (14)	0.0006 (15)	−0.0366 (15)
C6	0.0595 (14)	0.0521 (15)	0.0765 (18)	−0.0246 (12)	−0.0003 (12)	−0.0214 (13)
C7	0.0485 (11)	0.0408 (12)	0.0518 (13)	−0.0144 (9)	−0.0039 (9)	−0.0131 (10)
C8	0.0395 (11)	0.0485 (13)	0.0529 (14)	−0.0165 (10)	0.0048 (9)	−0.0129 (11)
C9	0.0431 (11)	0.0435 (12)	0.0502 (13)	−0.0122 (9)	0.0076 (9)	−0.0170 (10)
C10	0.0413 (10)	0.0396 (12)	0.0387 (11)	−0.0126 (9)	0.0038 (8)	−0.0118 (9)
C11	0.0445 (10)	0.0427 (12)	0.0382 (11)	−0.0139 (9)	0.0063 (8)	−0.0181 (10)
C12	0.0420 (10)	0.0402 (12)	0.0466 (12)	−0.0128 (9)	0.0008 (9)	−0.0187 (10)
C13	0.0406 (10)	0.0442 (12)	0.0412 (12)	−0.0156 (9)	−0.0003 (8)	−0.0130 (10)
C14	0.0428 (11)	0.0607 (15)	0.0544 (14)	−0.0161 (10)	0.0038 (10)	−0.0246 (12)
C15	0.0602 (15)	0.122 (3)	0.096 (2)	−0.0189 (16)	0.0195 (14)	−0.071 (2)
C16	0.0544 (14)	0.083 (2)	0.0708 (18)	−0.0321 (13)	0.0091 (12)	−0.0245 (15)
C17	0.0471 (12)	0.0516 (14)	0.0571 (14)	−0.0153 (10)	0.0027 (10)	−0.0225 (12)
C18	0.0415 (10)	0.0388 (11)	0.0484 (13)	−0.0139 (9)	0.0070 (9)	−0.0204 (10)
C19	0.0500 (12)	0.0555 (15)	0.0540 (15)	−0.0084 (11)	0.0060 (10)	−0.0182 (12)
C20	0.0670 (16)	0.0658 (18)	0.0583 (16)	−0.0065 (13)	−0.0052 (12)	−0.0077 (14)
C21	0.0595 (15)	0.0540 (16)	0.0779 (19)	−0.0032 (12)	−0.0184 (13)	−0.0176 (14)
C22	0.0443 (13)	0.0620 (17)	0.100 (2)	−0.0003 (12)	0.0017 (13)	−0.0358 (16)
C23	0.0546 (13)	0.0544 (15)	0.0657 (16)	−0.0128 (11)	0.0142 (11)	−0.0268 (13)
C24	0.0433 (13)	0.091 (2)	0.094 (2)	−0.0241 (13)	0.0203 (13)	−0.0373 (18)

### Geometric parameters (Å, º)

**Table:**

F1—C21	1.368 (3)	C10—C11	1.514 (3)
O1—C13	1.368 (2)	C11—C12	1.514 (3)
O1—C1	1.398 (2)	C11—C18	1.531 (3)
O2—C8	1.369 (2)	C11—H11	0.9800
O2—C24	1.424 (3)	C12—C13	1.350 (3)
N1—C14	1.293 (3)	C12—C17	1.425 (3)
N1—C13	1.359 (2)	C14—H14	0.9300
N2—C14	1.325 (3)	C15—H15A	0.9600
N2—C16	1.448 (3)	C15—H15B	0.9600
N2—C15	1.450 (3)	C15—H15C	0.9600
N3—C17	1.146 (3)	C16—H16A	0.9600
C1—C10	1.352 (3)	C16—H16B	0.9600
C1—C2	1.414 (3)	C16—H16C	0.9600
C2—C3	1.409 (3)	C18—C19	1.380 (3)
C2—C7	1.420 (3)	C18—C23	1.387 (3)
C3—C4	1.361 (3)	C19—C20	1.379 (3)
C3—H3	0.9300	C19—H19	0.9300
C4—C5	1.396 (3)	C20—C21	1.350 (4)
C4—H4	0.9300	C20—H20	0.9300
C5—C6	1.355 (3)	C21—C22	1.361 (4)
C5—H5	0.9300	C22—C23	1.390 (3)
C6—C7	1.415 (3)	C22—H22	0.9300
C6—H6	0.9300	C23—H23	0.9300
C7—C8	1.424 (3)	C24—H24A	0.9600
C8—C9	1.358 (3)	C24—H24B	0.9600
C9—C10	1.422 (3)	C24—H24C	0.9600
C9—H9	0.9300		
			
C13—O1—C1	118.87 (16)	C17—C12—C11	118.11 (18)
C8—O2—C24	116.89 (18)	C12—C13—N1	122.48 (19)
C14—N1—C13	119.91 (19)	C12—C13—O1	121.15 (17)
C14—N2—C16	121.1 (2)	N1—C13—O1	116.32 (18)
C14—N2—C15	121.8 (2)	N1—C14—N2	122.4 (2)
C16—N2—C15	116.9 (2)	N1—C14—H14	118.8
C10—C1—O1	122.70 (18)	N2—C14—H14	118.8
C10—C1—C2	122.93 (18)	N2—C15—H15A	109.5
O1—C1—C2	114.36 (18)	N2—C15—H15B	109.5
C3—C2—C1	123.11 (18)	H15A—C15—H15B	109.5
C3—C2—C7	119.0 (2)	N2—C15—H15C	109.5
C1—C2—C7	117.87 (19)	H15A—C15—H15C	109.5
C4—C3—C2	120.6 (2)	H15B—C15—H15C	109.5
C4—C3—H3	119.7	N2—C16—H16A	109.5
C2—C3—H3	119.7	N2—C16—H16B	109.5
C3—C4—C5	120.6 (2)	H16A—C16—H16B	109.5
C3—C4—H4	119.7	N2—C16—H16C	109.5
C5—C4—H4	119.7	H16A—C16—H16C	109.5
C6—C5—C4	120.4 (2)	H16B—C16—H16C	109.5
C6—C5—H5	119.8	N3—C17—C12	177.0 (2)
C4—C5—H5	119.8	C19—C18—C23	117.9 (2)
C5—C6—C7	121.1 (2)	C19—C18—C11	121.06 (18)
C5—C6—H6	119.4	C23—C18—C11	121.02 (19)
C7—C6—H6	119.4	C18—C19—C20	121.8 (2)
C6—C7—C2	118.3 (2)	C18—C19—H19	119.1
C6—C7—C8	122.9 (2)	C20—C19—H19	119.1
C2—C7—C8	118.84 (19)	C21—C20—C19	118.4 (3)
C9—C8—O2	124.9 (2)	C21—C20—H20	120.8
C9—C8—C7	120.82 (18)	C19—C20—H20	120.8
O2—C8—C7	114.24 (19)	C20—C21—C22	122.8 (2)
C8—C9—C10	120.8 (2)	C20—C21—F1	118.8 (3)
C8—C9—H9	119.6	C22—C21—F1	118.4 (2)
C10—C9—H9	119.6	C21—C22—C23	118.4 (2)
C1—C10—C9	118.72 (19)	C21—C22—H22	120.8
C1—C10—C11	121.56 (17)	C23—C22—H22	120.8
C9—C10—C11	119.67 (18)	C18—C23—C22	120.8 (2)
C10—C11—C12	108.81 (16)	C18—C23—H23	119.6
C10—C11—C18	110.34 (16)	C22—C23—H23	119.6
C12—C11—C18	112.23 (16)	O2—C24—H24A	109.5
C10—C11—H11	108.5	O2—C24—H24B	109.5
C12—C11—H11	108.5	H24A—C24—H24B	109.5
C18—C11—H11	108.5	O2—C24—H24C	109.5
C13—C12—C17	117.84 (18)	H24A—C24—H24C	109.5
C13—C12—C11	123.71 (18)	H24B—C24—H24C	109.5
			
C13—O1—C1—C10	10.3 (3)	C9—C10—C11—C12	166.21 (18)
C13—O1—C1—C2	−170.25 (17)	C1—C10—C11—C18	107.5 (2)
C10—C1—C2—C3	179.1 (2)	C9—C10—C11—C18	−70.2 (2)
O1—C1—C2—C3	−0.4 (3)	C10—C11—C12—C13	18.4 (3)
C10—C1—C2—C7	−0.7 (3)	C18—C11—C12—C13	−104.0 (2)
O1—C1—C2—C7	179.76 (17)	C10—C11—C12—C17	−168.54 (18)
C1—C2—C3—C4	179.8 (2)	C18—C11—C12—C17	69.1 (2)
C7—C2—C3—C4	−0.4 (3)	C17—C12—C13—N1	−3.0 (3)
C2—C3—C4—C5	0.2 (4)	C11—C12—C13—N1	170.10 (18)
C3—C4—C5—C6	−0.2 (4)	C17—C12—C13—O1	179.69 (18)
C4—C5—C6—C7	0.4 (4)	C11—C12—C13—O1	−7.2 (3)
C5—C6—C7—C2	−0.6 (4)	C14—N1—C13—C12	178.0 (2)
C5—C6—C7—C8	−179.9 (2)	C14—N1—C13—O1	−4.6 (3)
C3—C2—C7—C6	0.6 (3)	C1—O1—C13—C12	−8.2 (3)
C1—C2—C7—C6	−179.56 (19)	C1—O1—C13—N1	174.29 (17)
C3—C2—C7—C8	179.9 (2)	C13—N1—C14—N2	175.5 (2)
C1—C2—C7—C8	−0.2 (3)	C16—N2—C14—N1	−1.0 (4)
C24—O2—C8—C9	3.7 (3)	C15—N2—C14—N1	−175.6 (2)
C24—O2—C8—C7	−176.6 (2)	C10—C11—C18—C19	−89.7 (2)
C6—C7—C8—C9	179.6 (2)	C12—C11—C18—C19	31.8 (3)
C2—C7—C8—C9	0.3 (3)	C10—C11—C18—C23	87.5 (2)
C6—C7—C8—O2	−0.1 (3)	C12—C11—C18—C23	−151.0 (2)
C2—C7—C8—O2	−179.39 (18)	C23—C18—C19—C20	1.1 (4)
O2—C8—C9—C10	−179.8 (2)	C11—C18—C19—C20	178.4 (2)
C7—C8—C9—C10	0.5 (3)	C18—C19—C20—C21	−0.6 (4)
O1—C1—C10—C9	−178.99 (17)	C19—C20—C21—C22	−0.8 (4)
C2—C1—C10—C9	1.6 (3)	C19—C20—C21—F1	179.0 (2)
O1—C1—C10—C11	3.3 (3)	C20—C21—C22—C23	1.7 (4)
C2—C1—C10—C11	−176.16 (18)	F1—C21—C22—C23	−178.2 (2)
C8—C9—C10—C1	−1.4 (3)	C19—C18—C23—C22	−0.2 (3)
C8—C9—C10—C11	176.33 (19)	C11—C18—C23—C22	−177.5 (2)
C1—C10—C11—C12	−16.1 (3)	C21—C22—C23—C18	−1.1 (4)

### Hydrogen-bond geometry (Å, º)

Cg1, Cg2 and Cg3 are the centroids of the C18–C23, C2–C7 and C1,C2,C7–C10 rings, respectively.

**Table:**

*D*—H···*A*	*D*—H	H···*A*	*D*···*A*	*D*—H···*A*
C23—H23···N3^i^	0.93	2.62	3.542 (3)	171
C5—H5···*Cg*1^ii^	0.93	2.79	3.670 (3)	159
C15—H15*B*···*Cg*2^iii^	0.96	2.93	3.732 (3)	142
C16—H16*C*···*Cg*3^iii^	0.96	2.91	3.589 (3)	129

Symmetry codes: (i) −*x*+1, −*y*+1, −*z*+1; (ii) *x*, *y*−1, *z*; (iii) *x*+1, *y*, *z*.
